# Follicular Lymphoma: A Clinicopathological Analysis from a Tertiary Care Institute in Southern India

**DOI:** 10.4084/MJHID.2016.060

**Published:** 2016-11-01

**Authors:** Mary Theresa Sylvia, Biswajit Dey, Debdatta Basu, Sajini Elizabeth Jacob, Rakhee Kar, Biswajit Dubashi

**Affiliations:** 1 Department of Pathology, Jawaharlal Institute of Postgraduate Medical Education and Research, Pondicherry, India; 2 Department of Medical Oncology, Jawaharlal Institute of Postgraduate Medical Education and Research, Pondicherry, India

## Abstract

**Introduction:**

Follicular lymphoma (FL) is an indolent lymphoproliferative disorder of B-cells with variable clinical behavior. It is the second most common subtype of Non-Hodgkin lymphoma in western countries but reported to have a lower incidence in Asia.

**Materials and methods:**

Cases of FL diagnosed in the Department of Pathology of our Institute from January 2009 to June 2015 were included in the study. The clinicopathological parameters including staging, histological details, and immunohistochemical markers CD20, CD10 and BCL-2 were recorded in all the cases.

**Results:**

Of the 497 cases of Non-Hodgkin Lymphoma reported during the study period, 36 (7.2%) cases were follicular lymphoma. The mean age was 50 years with male to female ratio of 3.2:1. Grade 1/2 was seen in 70% cases. 22 % cases had low grade with high proliferation index (Ki67 > 40%). Granulomatous response was seen in two cases. Diffuse large cell lymphoma component was present in four cases. Bone marrow involvement and peripheral blood spill were seen in 12 (37.5%) and six cases (18.8%) respectively. 72% cases were in stage 3 or 4.

**Conclusion:**

The incidence of FL was lower in our study than other Indian studies. FL presented in the elderly, with male predominance and disseminated stage. The study highlights features of low grade with high proliferation index, granulomatous response, leukemic involvement, and transformation to high grade lymphoma.

## Introduction

Follicular lymphoma (FL) is an indolent B-cell lymphoma of germinal center origin. The translocation t(14,18) causes up regulation of *BCL-2* gene which leads to the inhibition of apoptosis of the germinal center cells. Therapy ranges from observation to chemotherapy. It is important to identify the high risk cases among this heterogeneous group with variable clinical behavior which requires intensive treatment unlike the low risk group. Recently grade 3b,^1^ low grade with high proliferation index^2^ and follicular lymphoma with granulomatous response^3^ are considered to behave similar to large cell lymphoma and thereby warrants intensive therapeutic regimens.^4^

## Materials and Methods

Cases of follicular lymphoma were collected from the archives of the Department of Pathology, of our Institute over a period of six years six months (January 2009 to June 2015). A total 36 cases of FL was retrieved during this period of six years six months. The age, gender, presenting complaints, fine needle aspiration cytology (FNAC), histological features along with grade, proliferation index, bone marrow, and peripheral blood involvement were noted. Grading was done using the world health organization (WHO) criteria. A panel of immunohistochemical markers CD 10, CD 20, BCL-2, CYCLIN D1 and CD5 was done in all the cases. One of the cases was negative for BCL-2, so BCL-6 was done in that case. CD 23 was performed in 11 cases and Ki-67 in 23 cases. Proliferation index was calculated as a percentage of positive cells in 10 random fields using the MIB-1(Ki-67) antibody more than 40% Ki-67 index was taken as the cut-off for high proliferation index. The staging was done according to the modified Ann-Arbor staging system. Clinical details, treatment history and follow-up as available were also collected.

## Results

### Clinical features

Follicular lymphoma constituted 7.2% (36 cases) of the 497 cases of Non-Hodgkin Lymphoma (NHL) reported during six years six months (January 2009 to June 2015). The mean age of patients with follicular lymphoma was 50 (Range 30–75 years). There was male predominance with M: F ratio of 3.2:1. Lymph node involvement was present in 35 cases, and one case had an extranodal (intestine) presentation. The clinical presentation is summarized in Table 1. Fine needle aspiration cytology was available for six cases which were reported as reactive in three cases, NHL (one case), large cell lymphoma (one case) and small lymphocytic lymphoma (one case). Staging done for 32 cases showed six cases (19%) to be stage 1, three (9%) stage 2, nine (28%) stage 3 and 14 cases (44%) stage 4. Post-mortem biopsies of one patient who had expired during evaluation showed infiltration of the liver and lung by follicular lymphoma cells.

### Histopathology

**Figure 1:**
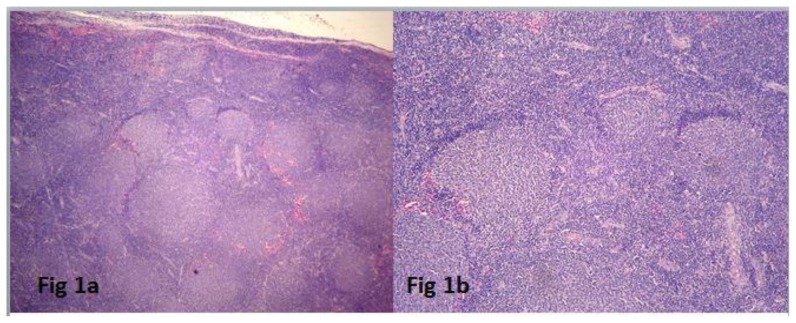
**a:** Low power view of lymph node showing follicular architecture. (H & E 100X). **b:** High power view of the follicles showing lack of apoptosis and tingible body macrophages in the germinal center. (H & E 400X).

**Figure 2:**
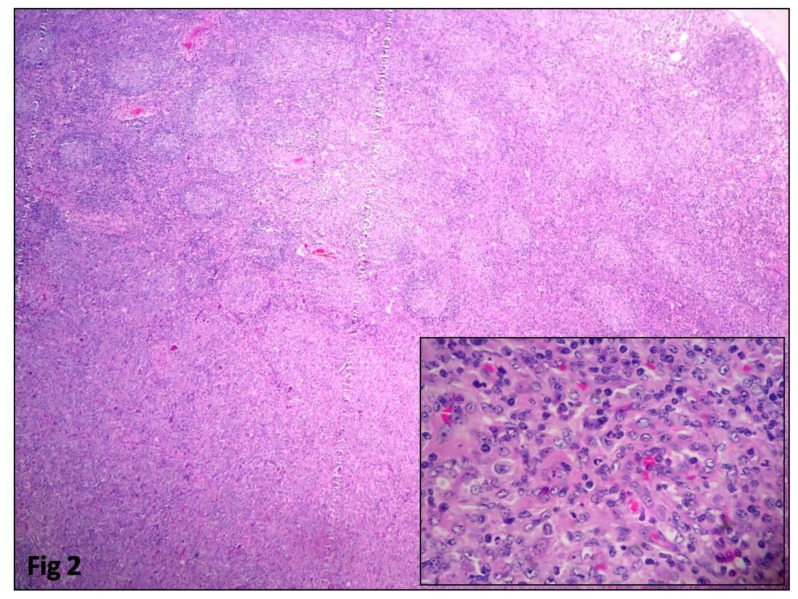
Composite histology of Follicular lymphoma transformation to large cell lymphoma. (H & E 100X). The inset shows high power view of the large cell lymphoma component. (H & E 400X).

All the cases had effacement of the lymph node by follicular architecture with follicles of similar size present in the cortex and medulla (Figure 1a). Tangible body macrophages were absent. High power view showed an admixture of centrocytes and centroblasts (Figure 1b). The percentage of centroblasts varied according to the grade. Grading was done in 31 cases. According to the WHO criteria for grading, eight cases (26%) were grade 1, 14 cases (45%) grade 2, six cases (19%) grade 3a and three cases (10%) grade 3b. The granulomatous response was seen in two cases. Follicular lymphoma with Diffuse large B cell lymphoma (DLBCL) arising in follicular lymphoma was observed in four cases (Figure 2). Composite histology was evident in two cases, and one was a relapse with partial transformation after two years. Clinicopathologic features are summarized in Table 2.

### Bone marrow involvement

**Figure 3:**
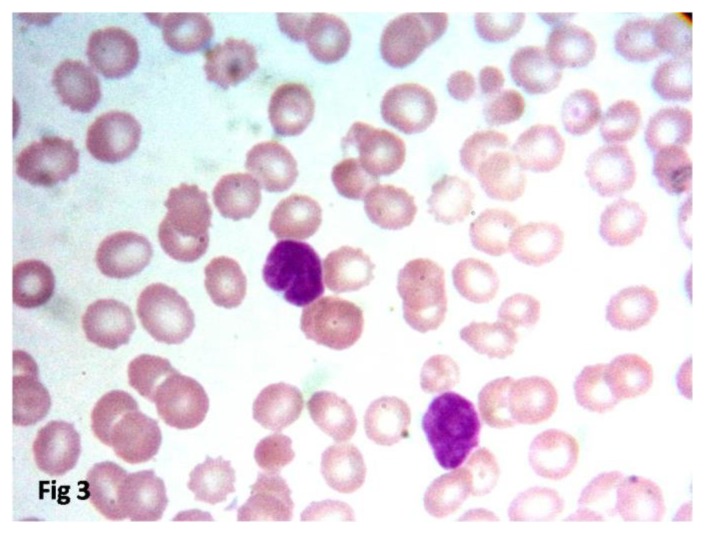
Peripheral smear showing follicular lymphoma cells. (Leishman 400X)

Bone marrow was available for 32 cases. Bone marrow was involved in 12 cases (37.5%) and six cases (18.8%) had peripheral blood spill (Figure 3). Bone marrow biopsy detected three cases with focal involvement by FL which was missed on aspirate smears. All the 12 cases showed focal paratrabecular structure either as a single pattern or mixed. The diffuse pattern of bone marrow involvement was seen in five cases and, the nodular and interstitial patterns were seen in four cases along with the focal paratrabecular localization. Single centrally located reactive lymphoid nodule was present in one patient. Bone marrow involvement is summarized in Table 3.

### Immunohistochemistry

**Figure 4:**
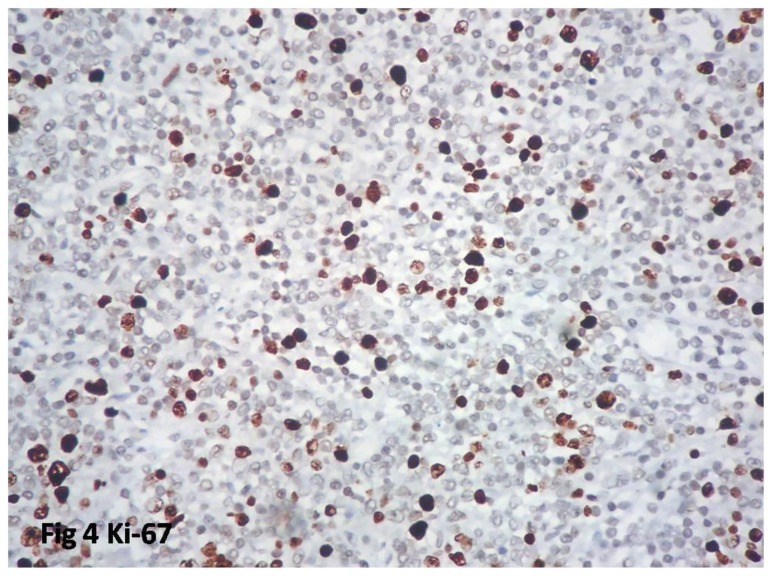
Ki-67 showing high proliferation index in low grade follicular lymphoma.(IHC 400 X).

All the cases were positive for CD 10 and CD 20; 35 cases for BCL-2, one case, BCL-2 negative was positive for BCL-6; all cases were negative for CYCLIN D1 and CD5. CD 23 was made in 11 cases of which three were diffusely positive, and three had stained the follicular dendritic cells, and five were negative. The proliferation index Ki-67 was performed in 23 cases. The range was from 10–60%. Low grade with high proliferation index was observed in five cases (22%) (Figure 4). Proliferation index is summarized in Table 4.

### FLIPI (Follicular lymphoma international prognostic index)

The FLIPI scoring could be done for 12 cases. The score was low in three cases (25%), intermediate in five (42%) and four (33%) were in the high risk category. The scoring is shown in Table 5.

### Follow up

**Figure 5:**
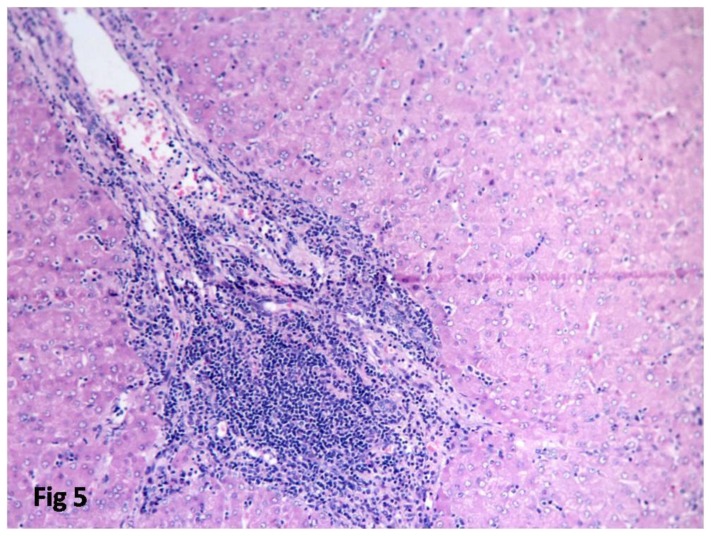
Postmortem liver biopsy showing portal infiltration by follicular lymphoma cells. (H & E 400X).

Follow up was available for 12 cases. Relapse was seen in six cases and five cases were in remission. The median relapse free time was 1.5 years. Two patients developed sensory neuropathy secondary to therapy. Leukemic infiltration of liver, lung, and spleen occurred in one patient who expired (Figure 5).

## Discussion

### Epidemiology

Follicular lymphomas are a heterogeneous group with regional variations in incidence. It is the second most common subtype of Non Hodgkin lymphoma in western countries constituting 20–25 % but reported to have a lower rate in Asia.^5^ Our incidence is lower (7.2%) compared to the previous Indian studies by Naresh et al., who had reported an incidence of 12.6%^6^ and Mondal et al. reported it to be 19.3% in eastern India.^7^ The average age of occurrence was 50 years in the present study. Literature quotes the median age of follicular to be a decade higher (59–60 years) in the developed countries.^8^,^9^ Among the Indian studies, Mondal et el had reported similarmean age of 51 years for follicular lymphoma from eastern India,^7^ Bharadwaj et al found the incidence to be a decade lower (41 years) in Uttarakand, India^10^ and Sharma et al had reported a bimodal occurrence (31–40 years and 51–60 years) in northern part of India.^11^ Western studies have documented female predominance^8^,^9^ but in our study, there was male predominance similar to the previous India studies. It is indolent and higher proportion of patients manifest in disseminated stage (3& 4)^8^,^9^ as in our series.

### Histopathology

#### FL with granulomas

The epithelioid granulomatous response was seen in two cases. Kojima et al. analyzed 50 cases of follicular and large cell lymphomas with granulomatous response and concluded it to be a separate entity presenting at an older age, bulkier disease and prognosis similar to DLBCL and inferior to follicular lymphoma. Hence he concluded this to be a separate entity of centroblastic/centrocytic lymphoma with epithelioid cell response.^3^ In contrast both our cases presented at an average age of 50 years not higher than the other cases, histological grade 2 and low proliferation index. One of them had bone marrow involvement. However, the number of cases is small to draw any conclusion.

### Transformation

Histological transformation of follicular lymphoma to aggressive variants most commonly DLBCL and rarely Burkitt or lymphoblastic lymphoma carries a poor prognosis. The frequency of transformation ranges from 10 to 60 % in the literature.^1^ It is in the lower range (11.1%) in our study. The current gold standard definition of transformation is the diffuse effacement of follicular architecture by an increase in large cells, histologically proven and clonally confirmed. Some authors require six months interval between the initial diagnosis of FL and DLBCL to establish transformation. The simultaneous presence of both features suggests but does not establish the diagnosis.^1^ Composite histology of DLBCL and FL at the initial diagnosis was present in two cases, and one case was a relapse of FL after two years with transformation to DLBCL. It is important to differentiate DLBCL from follicular lymphoma with diffuse architecture and grade 3b FL. FL with diffuse structure shows a mixture of centrocytes and centroblasts and lacks sheets of large cells. FL grade 3b has solid sheets of centroblasts (> 15 centroblasts/0.159 mm^2^) whereas DLBCL with FL component has diffuse area with solid sheets of centroblasts outside histologically or immunophenotypically (CD21, CD23+ FDC) recognizable follicles. DLBCL rising in FL is usually of germinal center phenotype^1^ as in our study.

The risk of transformation was higher in patients presenting with the bulkier disease, advanced stage, B symptoms and high FLIPI scores.^1^ We do not have data regarding the initial presentation in the relapse case.

The clinical features at transformation include old age i.e. more than 60 years, a discordant growth of lymph nodes, increase in the stage, the appearance of B symptoms and extranodal involvement.^1^ The average age of our four cases was 60 years, the advanced stage frequent, in one patient with primitive extranodal localization and the relapse of the disease was in the lymph nodes.

### Bone marrow

Bone marrow involvement in our study was 37.5%. Three cases were missed on aspirate and diagnosed on biopsy reinforcing the need for biopsy for staging. All cases had concordant involvement. Bone marrow involvement has been included as one of the prognostic factors in the FLIPI scoring system. Paratrabecular localization has been described as most common and characteristic of follicular lymphomas.^12^ We found it in all the 12 (100%) cases. Single centrally located reactive lymphoid nodule was seen in one case causing a diagnostic difficulty. Differentiation of reactive nodule from the follicular pattern of involvement by FL requires a synopsis of criteria. The topographical location (central perivascular in reactive, paratrabecular in FL), peripheral infiltration “Indian file” like (well defined border in reactive) and dense reticulin fibres help the morphological differentiation in FL. Use of immunohistochemistry is also critical. Both can have a mixture of T and B cells and can express BCL-2.^13^

Bone marrow involvement was more common in grade 1 and 2 rather than grade 3 cases. Hence bone marrow involvement appears early. This is explained by Kluin et al. as a bidirectional migration of the tumor cells both in the lymph nodes and marrow at the beginning of the disease process.^14^

### Peripheral blood involvement

Peripheral blood involvement was seen in 18.8 % of our patients, which is higher than the previous studies.^15^ Circulating cells of follicular lymphoma are seen in many cases, but leukemic involvement ranges from 4–23%.^16^ Four of our cases had a detectable leukemic phase at the time of diagnosis. Few circulating lymphoma cells do not affect the prognosis, but leukemic spread and high total counts have an adverse prognosis. Sarkozy et al. reported 7.4 % of their cases to have leukemic involvement and had shorter progression free and overall survival compared to a group of follicular lymphoma without a leukemic spill. More than 4×10^9^/L circulating lymphoma cells was found to have a bad prognosis.^17^ In the present study out of the four patients in leukemic phase one patient expired, the second one had an initial response to chemotherapy, and the other two were lost to follow up.

### Proliferation index

The proliferation rate of lymphomas is assessed using the MIB 1 antibody (Ki-67). Xin He et al. had done a meta-analysis of Ki-67 index in lymphomas and found higher proliferation to be associated with inferior overall survival and disease free survival rate.^18^ But the study has not highlighted the importance in follicular lymphomas. Low grade follicular lymphomas (grade 1 &2) with high proliferation index (Ki-67) have been reported recently.^2^ Ki-67 index of more than 40 % is considered as high proliferation index.^2^ This subgroup has been found to behave as higher grade lymphomas/DLBCL and requires high dose chemotherapy. When estimating the Ki-67 index, it is important to avoid areas of normal germinal centers in a partially effaced node and areas of transformation to high grade lymphoma which usually have high proliferation index. In the present study, 22% of the cases were of low grade with high proliferation index as compared to the western literature of 18%.^18^ The higher percentage of our cases may be due to racial and geographical variation. Wang et al. had also proved high proliferation index as a poor prognostic marker in follicular lymphoma.^19^ Hence it is important to identify this subgroup which needs intensive therapy. All our five cases were in an advanced stage; one had progressive disease and one in remission. Other 3 cases were lost to follow-up.

### CD 23

CD 23 is a low affinity receptor for IgE, present in follicular dendritic cells (FDC) and promoting survival of germinal center B cells. It is positive in chronic lymphocytic leukemia. Recently CD 23 positivity in neoplastic cells has been described in follicular lymphomas. Olteanu et al. have reported 70% of their cases of follicular lymphomas to be positive for CD23. It was associated with inguinal lymphadenopathy, lower grade, and a better prognosis compared to the negative group.^20^ In our series CD23 was done in 11 cases of which the three positive cases were of lower grade. However one of the cases of the inguinal lymph node was negative in contrast to previous studies which have reported an association with inguinal lymph nodes. 20 The concept of tumor microenvironment in FL has gained importance. The FDC and T regulatory cells play a significant role in FL in the lymph node as well as bone marrow colonisation.^21^ Hence it is mandatory to study it in detail.

### BCL-6

BCL-6 located in 3q27 functions as a transcriptional repressor of germinal center B cells. It is highly expressed in FL, but translocation is present in 6.4% to 14.3% of FL.^22^ It coexists with *BCL-2* translocation in half of the cases. Akasaka et al. have reported BCL-6 positivity as a marker of genomic instability and early transformation.^22^ One of our cases was negative for BCL-2, so we did BCL-6 which was positive, but not routinely performed in all cases.

### Prognostic scoring

The FLIPI scoring distribution is comparable with the previous study by Solal-Celigny et al. (2004)^23^ as shown in Table 5. However, the numbers of cases are inadequate for further survival analysis.

## Conclusion

The incidence of follicular lymphomas is lower in Southern India as compared to western countries and other parts of India and, has a male predominance. Features like grades 3b, follicular lymphomas with the granulomatous response, low grade with high Ki-67 index are highlighted. Involvement of bone marrow and peripheral blood is high in follicular lymphomas. It is mandatory to do Ki-67, CD 23 and BCL-6 in all cases of follicular lymphomas, and *P53* in transformed cases. Hence it is important to identify the high risk cases in this low grade group which can be treated with high dose regimens or experimental therapies.

## Figures and Tables

**Table 1:** The clinicopathological profile of 36 cases of follicular lymphoma.

Parameters	No. Of Cases (%)
Nodal	35 (97%)
Extranodal (intestine)	1 (3%)
Hepatosplenomegaly	6 (17%)
Ascites	2 (6%)
Pleural Effusion	2 (6%)
Lymphedema (Inguinal node)	2 (6%)
Bleeding per rectum	1 (3%)
Secondary in a case of carcinoma bladder	1 (3%)

**Table 2:** The grade and clinical stage of the cases

Parameters	No. Of Cases (%)
Stage 1	6/32 (19%)
Stage 2	3/32 (9%)
Stage 3	9/32 (28%)
Stage 4	14/32 (44%)
Grade 1	8/31 (26%)
Grade 2	14/31 (45%)
Grade 3a	6/31 (19%)
Grade 3b	3/31 (10%)

Note: Staging was done in 32 and grading in 31 of the 36 cases.

**Table 3:** Pattern of bone marrow involvement in 12 cases.

Parameters	No. Of Cases (%)
Reactive lymphoid nodule	1/12 (8.3%)
Paratrabecular pattern	12/12 (100%)
Diffuse	5/12 (41.7%)
Nodular & interstitial	4/12 (33.3%)

**Table 4:** Proliferation index (Ki-67) in 23 cases at diagnosis.

Parameters	No. Of Cases (%)
<20 %	11/23 (48%)
21–40%	5/23 (22%)
41–60%	7/23 (30%)

**Table 5:** FLIPI scoring done in 12 cases.

No of risk factors	FLIPI score	No of cases (%)
0–1	Low	3/12 (25%)
2	Intermediate	5/12 (42%)
3 or >	High	4/12 (33%)
